# Differences in medical costs between TCM users and TCM nonusers in inpatients with thalassemia

**DOI:** 10.1186/s12913-023-09651-w

**Published:** 2023-06-14

**Authors:** Zhaoran Han, Hanlin Nie, Zhengwei Huang, Zegui Tuo, Sisi Chen, Yong Ma, Xuefeng Shi

**Affiliations:** 1 School of Management, Beijing University of Chinese Medicine, Beijing, China; 2 The Jockey Club School of Public Health and Primary Care, Faculty of Medicine, The Chinese University of Hong Kong, Shatin, Hong Kong SAR China; 3 China Health Insurance Research Association, Beijing, China

**Keywords:** Thalassemia, Traditional Chinese Medicine, Inpatient cost, Mainland China

## Abstract

**Background:**

Thalassemia has brought serious health threats and economic burdens to patients worldwide. There is no sovereign remedy for thalassemia, both conventional and Traditional Medicine (TM) methods have certain effects on this disease. As typical of TM, Traditional Chinese Medicine (TCM) has been widely used in the treatment of thalassemia. Previous studies mainly focused on conventional treatments for thalassemia and patients’ medical burden, but no research has examined the effects of TCM use on the economic burdens for thalassemia inpatients in mainland China. The main objective of this study is to compare the medical cost differences between TCM users and TCM nonusers, furtherly, we will discuss the role of TCM use in the treatment of thalassemia.

**Methods:**

We employed the 2010–2016 Medicare claims database provided by the China Health Insurance Research Association (CHIRA). Chi-square and Mann-Whitney tests were used to analyze the differences between TCM users and TCM nonusers. Multiple regression analysis was performed using the ordinary least squares method to compare the TCM users’ inpatient medical cost with TCM nonusers’, and to further examine the correlation between TCM cost, conventional medication cost and nonpharmacy cost for TCM users.

**Results:**

A total of 588 urban thalassemia inpatients were identified, including 222 TCM users and 366 TCM nonusers. The inpatient medical cost of TCM users was RMB10,048 (USD1,513), which was significantly higher than TCM nonusers (RMB1,816 (USD273)). Total inpatient cost for TCM users was 67.4% higher than those of TCM nonusers (P < 0.001). With confounding factors fixed, we found that the conventional medication cost and nonpharmacy cost were positively correlated with TCM cost.

**Conclusion:**

Total hospitalization expenses for TCM users were higher than TCM nonusers. Both the conventional medication cost and nonpharmacy cost of TCM users were all higher than TCM nonusers. We infer TCM plays a complementary role, rather than an alternative, in the treatment of thalassemia due to the lack of cooperative treatment guidelines. It is recommended that a cooperative diagnosis and treatment guidelines should be generated to balance the use of TCM and conventional medicine for treating thalassemia, so as to reduce the economic burdens on patients.

**Supplementary Information:**

The online version contains supplementary material available at 10.1186/s12913-023-09651-w.

## Background

Thalassemias are a group of inherited hematologic disorders caused by defects in the synthesis of one or more of the hemoglobin chains [1], α thalassemia and β thalassemia are most common clinically. There were about 399 million thalassemia carriers in 2019 worldwide [2], mainly in sub-Saharan Africa, the Mediterranean region, and East and South-East Asia, and thalassemia is becoming more common in Europe and North America due to migration factors [3, 4]. The increased morbidity of thalassemia has resulted in serious health threats and heavy economic burdens to the patients [5], especially in low and middle-income countries [4]. It was estimated that the global cost of treatment for thalassemia was approximately USD842 million in 2017 and is expected to increase by 7.9% from 2018 to 2026 [2]. In the 2017 Global Burden of Disease report, thalassemia has resulted in 582,000 disability-adjusted life-years (DALYs) [6]. Given the importance of preventing hemoglobinopathy, WHO has declared hemoglobinopathy control, especially β-thalassemia, a priority for the developing world [7].

Thalassemia is prevalent in tropical and subtropical areas regions [3]. Due to geographical and genetic factors, thalassemia is most common in southern regions of China such as Guangdong, Guangxi, Hainan, and other provinces [8]. A meta-analysis displayed that the combined overall prevalence of α, β, and α + β was 7.88%, 2.21%, and 0.48%, respectively [9]. Lin et al. collected data from 45 patients with thalassemia in Guangdong Province in 2012 and found that the per capita annual direct economic burden of patients was RMB43,058.66 (USD6,482), the per capita annual indirect economic burden was RMB20,474.51 (USD3,082), and the per capita intangible economic burden was RMB302,466.67 (USD45,536) [10]. The annual direct economic burden alone exceeded the per capita disposable income of Guangdong Province (RMB30,226.71(USD4,551)) in that year. In 2016, a study on the cost of rare diseases in Fujian Province showed that the average annual medical cost of thalassemia patients was RMB79,200 (USD11,924), of which the out-of-pocket cost was RMB44,600 (USD6,715), and the proportion of households with catastrophic expenditure was as high as 94.12% [11], the annual treatment cost exceeded the per capita disposable income of RMB36,014.26 (USD5,422) in Fujian Province. The economic burdens and health threats faced by patients in China are in a very serious situation.

The recommended treatment for thalassemia patients with severe conditions includes regular lifelong blood transfusions and iron chelation, and there is no sovereign remedy for patients with severe thalassemia except hematopoietic stem cell transplantation [12]. Most patients could hardly afford the highly expenses, so they had to struggle to seek other approaches, such as complementary and alternative medicine (CAM). A survey conducted in Iran showed that 68.5% of thalassemia respondents had used CAM at least once in their lifetime [13]. In Turkey, 82.5% of parents of children with thalassemia reported that they had used multiple CAM to treat their children [14]. The proportion of people with thalassemia in Malaysia who use CAM even exceeds cancer patients [15, 16]. Traditional Chinese medicine (TCM) is typical of CAM and plays an important role in the healthcare system both in China and in many other East Asian countries [17]. The Chinese government has always attached great importance to the development of TCM, and in 2016 issued the *Outline of Strategic Planning for the Development of Traditional Chinese Medicine (2016–2030)*, the government emphasized “attached equal importance to TCM and Western medicine”, and encouraged the joint research of TCM and western medicine for major and difficult diseases, and form a unique strategy for integrating TCM and western medicine.

With the broad use of TCM, more and more research have emerged to examine the role of TCM on disease treatment and the influence of using TCM on patients’ economic burdens. Some studies showed that the treatment of thalassemia with TCM can improve the hematopoietic function, reduce the damage of red blood cells, alleviate the symptoms of anemia, and enhance the quality of life [18–20]. Lin et al. found that in Dementia, TCM users could lower inpatient medical cost and length of stay compared to TCM nonusers [21]. Liao et al., using Taiwan’s 2005 Longitudinal Health Insurance Database, found that the TCM insurance cost was consistently lower than those covering biomedicine in patients with liver cancer [17]. Huang et al. concluded that TCM mainly played a complementary role to conventional medicine in the treatment of Chinese mainland ischemic stroke [22]. The above researches demonstrated the influence of TCM using either on treatment effect or on the economic burdens of other diseases. It is noteworthy that although TCM has been used in the treatment of thalassemia, there is no research illustrating the effect of TCM use on the economic burdens of thalassemia inpatients. However, as a significant approach for curing thalassemia, the role of TCM is worthy of attention, the economic burdens of TCM use on thalassemia inpatients need to examine. To explore the above issues, we used cross-sectional data from 2010 to 2016 for research, and further evaluate the correlation between TCM use and TCM cost.

## Materials and methods

### Data source

**Fig. 1:**
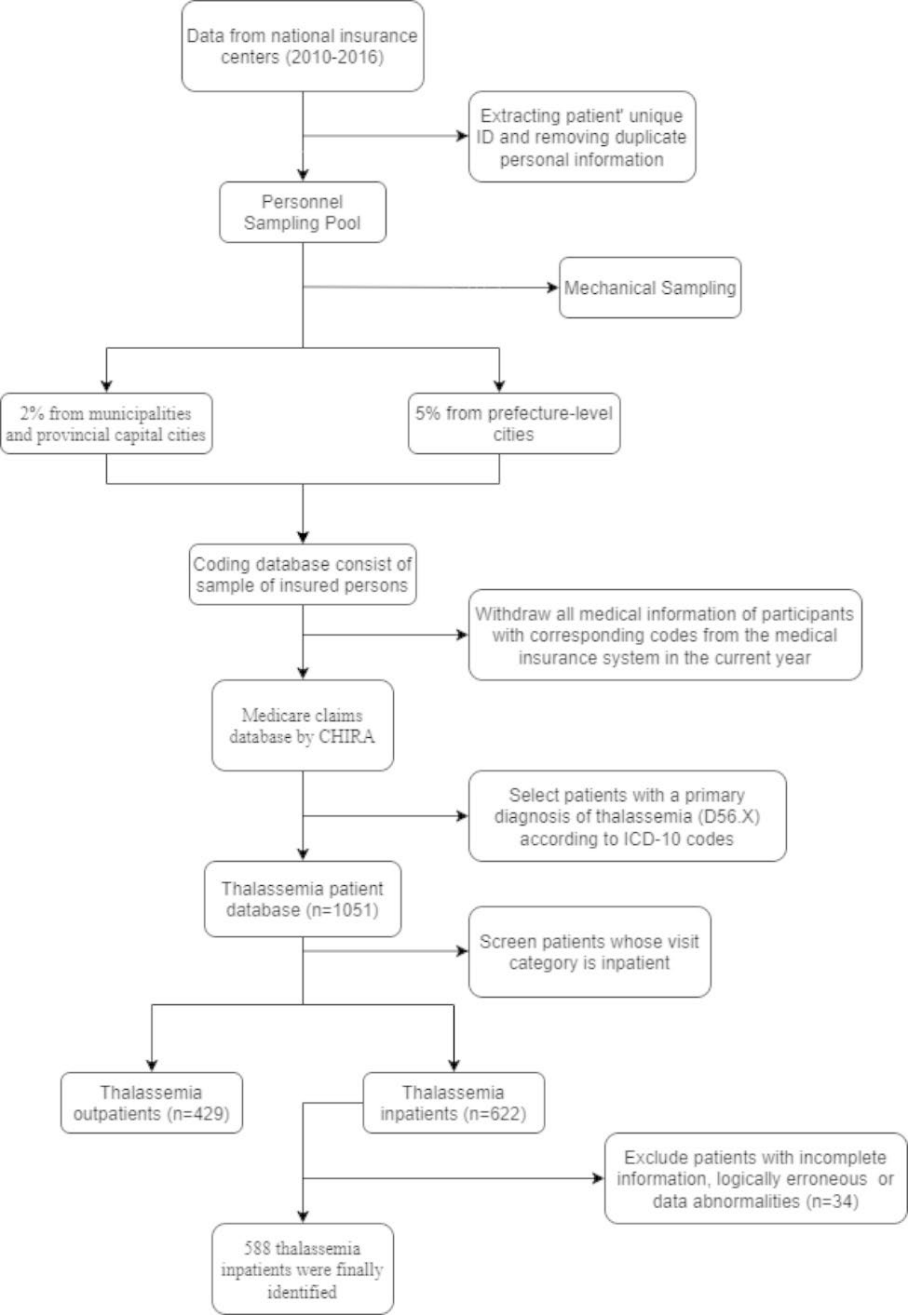
Sample selection process for thalassemia patients

The data was obtained from the Medicare claims database provided by China Health Insurance Research Association (CHIRA). The CHIRA database is a random sample of 5% data from Urban Employee Basic Medical Insurance (UEBMI) and Urban Resident Basic Medical Insurance (URBMI) schemes. UEBMI and URBMI covered more than 95% of the urban population in China [23]. CHIRA data is annually collected from local insurance centers in the selected areas of mainland China, at least 2% from municipalities and provincial capital cities, and 5% from prefecture-level cities. We can identify all medical services and patients’ expenditures based on the medicare claims database. The database is currently available for research between 2010 and 2016. According to the International Classification of Diseases, 10th Revision (ICD-10), we extracted thalassemia with a major diagnosis between 2010 and 2016 (D56.0, D56.1, D56.2, D56.3, D56.4, D56.9) part of the patient’s information, including the patient’s basic information (sex, birth date, insurance type, etc.), medical institution information (hospital level, region, etc.), healthcare service utilization, and healthcare expenditure details (length of stay, service items, medical costs, etc.). Patient data with incomplete information, logically erroneous, or data abnormalities were excluded. We identified a group of 2010–2016 cross-sectional data consisting of 588 thalassemia inpatients. The sample selection process is shown in detail in Fig. 1.

### Measures

According to the CHIRA claims database classification, TCM users in this research are defined as inpatients with thalassemia who use any one or more of the three TCM treatments of Chinese herbal medicine, Chinese patent medicine, and Chinese medicine injection (For detailed definitions of the three terms see Additional file 1). TCM nonusers are defined as inpatients who do not use any of the TCM treatments. The region is divided into east, central, and west regions according to geographic location and economic development. The number of hospitalizations is the number of patient ID code appearances. Manually label the number of comorbidities in the database as a measure of the disease severity of patients.

In terms of medical costs, the total inpatient medical costs are the sum of the total medication and nonpharmacy costs. The total medication costs include conventional medication and TCM costs. Nonpharmacy costs refer to expenses except medication costs, such as surgery, medical devices, medical services, etc. Patient data for multiple visits in the claims database is calculated by adding up the medical costs in each record based on the patient’s ID code, so the annual hospitalization costs can be obtained. The average exchange rate between USD and RMB in 2016 was adopted for currency value conversion: USD1 = RMB6.6423.

### Statistical analysis

The descriptive analysis section shows the demographic characteristics and inpatient medical cost with thalassemia. Categorical variables are expressed by percentages, and continuous variables are expressed by means or median with interquartile range (IQR). The Chi-square test and Mann-Whitney test are used to examine differences between TCM users and TCM nonusers. Data of cost usually have a positively skewed distribution, so in the regression analysis section, a logarithmic conversion is performed on medication cost. After adjusting for the confounding variables, ordinary least squares regression analysis to further examine the differences in inpatient medical cost between TCM users and TCM nonusers. Finally, two regression models are established with the logarithm of TCM cost as the dependent variable, the logarithm of conventional medication cost and the logarithm of nonpharmacy cost as independent variables respectively to explore the correlation between the TCM cost and the conventional medication cost as well as nonpharmacy cost. The regression result (β) has been transformed using the formula, *Coefficient* = e^β^−1. Statistical analyses were performed using STATA/MP 17.0, and two-sided *P* values less than 0.05 was considered statistically significant.

## Results

### Patient characteristics

As shown in Tables 1 and 588 hospitalized patients with thalassemia, of which 222 (37.8%) patients (TCM users) had used TCM during treatment. The median age of TCM users was significantly higher than TCM nonusers (P < 0.001). The proportion of male TCM nonusers (55.5%) was significantly higher than that of TCM users (39.2%), while that of women was the opposite (P < 0.001). In terms of insurance type, the majority of patients were from URBMI (77.9%), and there were only 10.1% of UEBMI-insured patients among TCM nonusers. Overall, 62.6% of patients were treated in tertiary hospitals. 45.1% of TCM users came from the west region and 41.4% came from the east region. 94.3% of TCM nonusers had no comorbidities, significantly higher than TCM users (71.6%) (P < 0.001). The average length of stay (ALOS) was 17.7 days for TCM users, significantly longer than 9.1 days for TCM nonusers (P < 0.001). The average number of hospitalizations per year for TCM users (3.6) is more than TCM nonusers (2.6).

**Table 1:** Sample characteristics of thalassemia inpatients

Characteristics		Overall	TCM nonusers	TCM users	*P*-value
Sex, *n* (%)	Male	290(49.3)	203(55.5)	87(39.2)	< 0.001
	Female	298(50.7)	163(44.5)	135(60.8)	
Age(years), median (IQR)		9(5–28)	7(4–12)	28(7–57)	< 0.001
Age group, *n* (%)	0-	185(31.5)	150(41.0)	35(15.8)	< 0.001
	5-	200(34.0)	152(41.5)	48(21.6)	
	15-	41(7.0)	25(6.8)	16(7.2)	
	25-	55(9.4)	20(5.5)	35(15.8)	
	35-	21(3.6)	6(1.6)	15(6.8)	
	45-	22(3.7)	5(1.4)	17(7.7)	
	55-	64(10.9)	8(2.2)	56(25.2)	
Insurance type, *n* (%)	UEBMI	130(22.1)	37(10.1)	93(41.9)	< 0.001
	URBMI	458(77.9)	329(89.9)	129(58.1)	
Hospital level, *n* (%)	Primary	36(6.1)	24(6.6)	12(5.4)	0.553
	Secondary	184(31.3)	119(32.5)	65(29.3)	
	Tertiary	368(62.6)	223(60.9)	145(65.3)	
Region, *n* (%)	East	250(42.5)	158(43.2)	92(41.4)	< 0.001
	Central	46(7.8)	16(4.4)	30(13.5)	
	West	292(49.7)	192(52.5)	100(45.1)	
Type of city, *n* (%)	County-level city	27(4.6)	24(6.6)	3(1.4)	< 0.001
	Prefecture-level city	357(60.7)	230(62.8)	127(57.2)	
	Provincial capital city	140(23.8)	62(16.9)	78(35.1)	
	Municipality	64(10.9)	50(13.7)	14(6.3)	
No of comorbidities, *n* (%)	0	504(85.7)	345(94.3)	159(71.6)	< 0.001
	1	38(6.5)	17(4.6)	21(9.5)	
	2+	46(7.8)	4(1.1)	42(18.9)	
ALOS		12.3	9.1	17.7	< 0.001
No of hospitalizations		3.0	2.6	3.6	0.086
Year, *n* (%)	2010	59(10.0)	50(13.7)	9(4.1)	< 0.001
	2011	74(12.6)	59(16.1)	15(6.8)	
	2012	101(17.2)	74(20.2)	27(12.2)	
	2013	108(18.4)	50(13.7)	58(26.1)	
	2014	31(5.3)	7(1.9)	24(10.8)	
	2015	128(21.8)	82(22.4)	46(20.7)	
	2016	87(14.8)	44(12.0)	43(19.4)	
Number of patients, *n* (%)		588(100.0)	366(62.2)	222(37.8)	

*P* values are based on the chi-square test and Mann-Whitney test; TCM: traditional Chinese medicine, UEBMI: Urban Employee Basic Medical Insurance scheme, URBMI: Urban Resident Basic Medical Insurance scheme, IQR: interquartile range; Municipality: municipality directly under the Central Government (i.e.Beijing, Shanghai, Tianjin, and Chongqing); ALOS: Average length of stay.

### Total inpatient cost between TCM users and TCM nonusers

We compared the total hospitalization costs of inpatients with different population characteristics. Overall, total inpatient costs of TCM users were RMB10,048 (USD1,513), significantly higher than TCM nonusers (RMB1,816 (USD273)) (P < 0.001).

As shown in Table 2, the total inpatient costs of TCM users aged 0–14 and 25–44, as well as TCM users without comorbidities, were significantly higher than those of TCM nonusers (P < 0.01). In terms of sex, insurance type, hospital level, region, type of city, and year, the total inpatient costs of TCM users were significantly higher than TCM nonusers (all P < 0.05).

**Table 2:** Total inpatient cost of TCM users and TCM nonusers

Characteristics		TCM nonusers		TCM users		*P*-value
		Median	IQR	Median	IQR	
Sex	Male	1780	1200–4818	12866	3405–29615	< 0.001
	Female	1882	1157–3868	8860	5602–23674	< 0.001
Age group	0-	1332	980–2365	5279	3065–14781	< 0.001
	5-	1955	1399–4753	18187	3311–34175	< 0.001
	15-	5517	2191–39513	6280	4590–16043	0.669
	25-	2264	1657–7818	8506	6350–13281	< 0.001
	35-	1869	1245–2372	11426	5781–28339	0.005
	45-	9583	5353–9624	15577	5426–27623	0.196
	55-	7542	3483–8634	10755	5524–27358	0.084
Insurance type	UEBMI	5353	1616–15168	10015	6323–23772	0.001
	URBMI	1763	1146–3691	10081	3906–28339	< 0.001
Hospital level	Primary	1108	356–1463	6610	3013–33689	< 0.001
	Secondary	1726	1191–3245	6704	4340–22881	< 0.001
	Tertiary	2084	1249–5609	10826	5781–26252	< 0.001
Region	East	2137	1203–5658	8683	5676–23542	< 0.001
	Central	3391	1118–12396	15789	4822–29366	0.016
	West	1747	1178–2950	10898	3906–26230	< 0.001
Type of city	County-level city	801	356–1227	1610	1581–16560	0.021
	Prefecture-level city	1929	1220–5553	11426	5602–27623	< 0.001
	Provincial capital city	2330	1245–5353	7346	4340–24568	< 0.001
	Municipality	1787	1329–2247	6914	3875–49102	< 0.001
No of comorbidities	0	1740	1149–3789	10826	4540–28464	< 0.001
	1	9354	2518–19320	11325	4987–22391	0.371
	2-	5694	2675–14499	8010	6323–12048	0.483
Year	2010	963	456–1484	2742	1714–6362	< 0.001
	2011	1389	930–1766	4007	1720–9293	< 0.001
	2012	1854	1151–3289	4898	2493–8645	< 0.001
	2013	2289	1392–7661	7778	5940–20403	< 0.001
	2014	3866	3062–7496	23723	11994–35524	0.005
	2015	3974	1780–14344	13104	5274–24575	< 0.001
	2016	2408	1606–19661	23411	5808–39810	< 0.001

*P* values are based on the Mann–Whitney test; TCM: traditional Chinese medicine; UEBMI: Urban Employee Basic Medical Insurance scheme; URBMI: Urban Resident Basic Medical Insurance scheme; IQR: Interquartile range. Municipality: municipality directly under the Central Government (i.e.Beijing, Shanghai, Tianjin, and Chongqing)

### Multiple regression analysis of total inpatient costs of TCM users and TCM nonusers

Table 3 shows a comparative model of the differences in multiple regression analysis of the total inpatient costs between TCM users and TCM nonusers. After controlling for the average length of stay (ALOS), sex, age, number of comorbidities, insurance type, hospital type, region, type of city, year, and number of hospitalizations, we found that TCM users had 67.4% (= exp^0.515^-1) higher medical costs than TCM nonusers (P < 0.001).

**Table 3:** Multiple regression analysis for total inpatient costs

Parameters		Coef.	*P* ≥ *z*	95% Wald confidence interval	
				Lower	Upper
Use of TCM	TCM user	0.515	< 0.001	0.360	0.670
ALOS		0.018	< 0.001	0.012	0.024
Sex	Female	-0.028	0.662	-0.153	0.098
Age		0.017	< 0.001	0.013	0.021
No of comorbidities		0.026	0.645	-0.084	0.135
Insurance type	URBMI	-0.060	0.561	-0.263	0.143
Hospital type	Secondary	0.216	0.167	-0.090	0.521
	Tertiary	0.392	0.014	0.081	0.704
Region	Central	-0.201	0.117	-0.453	0.050
	West	-0.221	0.004	-0.372	-0.071
Type of city	Prefecture-level city	0.405	0.028	0.044	0.765
	Provincial capital city	0.372	0.048	0.003	0.741
	Municipality	0.292	0.194	-0.149	0.733
Year	2011	0.247	0.070	-0.020	0.515
	2012	0.452	< 0.001	0.209	0.696
	2013	0.821	< 0.001	0.558	1.084
	2014	1.546	< 0.001	1.196	1.895
	2015	0.886	< 0.001	0.621	1.151
	2016	0.958	< 0.001	0.671	1.246
No of hospitalizations		0.096	< 0.001	0.074	0.118
_Cons		6.151	< 0.001	5.760	6.541

R-square = 0.7198 and adjusted R-square = 0.7099 in a multiple linear regression model that was adjusted for average length of stay, sex, age, number of comorbidities, insurance type, hospital type, region, type of city, year of treatment, and number of hospitalizations. The baseline represents the inpatient cost for a male who did not use any TCM with Urban Employee Basic Medical Insurance admitted to a primary hospital in a county-level city in the east region without any comorbidity. TCM: traditional Chinese medicine; UEBMI: Urban Employee Basic Medical Insurance scheme; URBMI: Urban Resident Basic Medical Insurance scheme; Municipality: municipality directly under the Central Government (i.e.Beijing, Shanghai, Tianjin, and Chongqing); ALOS: Average length of stay

### Composition of TCM users and TCM nonusers total Inpatient Medical costs

To further explore the reasons for the higher hospitalization costs among TCM users than among TCM nonusers, we compared the composition of the total inpatient costs between the two groups of populations. According to the classification of the insurance payment system, the total inpatient costs with thalassemia were divided into conventional medication costs, TCM costs, and nonpharmacy costs. Table 4 shows that the costs of TCM accounted for only 4.5% of the total inpatient medical costs, while the conventional medication costs and nonpharmacy costs of TCM users accounted for 39.9% and 55.7% of the total inpatient cost, respectively, which shows that the use of TCM is not the only reason for the higher costs of TCM users.

**Table 4:** Composition of medication and medical cost of TCM users and TCM nonusers

Variables		TCM nonusers	TCM users	*P*-value
Total medical cost	Median	1816	10,048	< 0.001
	IQR	1191–4288	4966–26,207	
Total medication cost	Median	464	2671	< 0.001
	IQR	17-1557	932–9664	
	Percentage	54.8	44.3	
Conventional medication cost	Median	469	2259	< 0.001
	IQR	19-1682	633–8455	
	Percentage	54.8	39.9	
TCM cost	Median	-	179	
	IQR	-	54–659	
	Percentage	-	4.5	
Nonpharmacy cost	Median	1304	5966	< 0.001
	IQR	760–2389	2679–13,576	
	Percentage	45.2	55.7	

*P* values are based on the Mann–Whitney test; TCM: traditional Chinese medicine; IQR: interquartile range.

### Multiple regression analysis to test the correlation between TCM cost and conventional medication cost and nonpharmacy cost

To further examine the reasons for the higher inpatient costs among TCM users, we generated two models to illustrate the correlation between TCM costs and conventional medication costs, as well as nonpharmacy costs (see Table 5 for details). We found that TCM costs was positively correlated with conventional medication costs and nonpharmacy costs. In other words, with the increase of the use of conventional medication and nonpharmacy diagnosis and treatment methods, the cost of TCM was also increasing, showing a trend of the same growth, indicating that the relationship between TCM and conventional medication and nonpharmacy treatment are not mutually alternative, and the role played by TCM is more of a supplement. There are two possible explanations. First, the disease severity of TCM users is more serious than TCM nonusers. Second, the lack of cooperative diagnosis and treatment guidelines resulted in failure of balancing the use of TCM and conventional medication.

**Table 5:** Multiple regression analysis for TCM cost

Parameters		Coef.	*P* > *z*	95% Wald confidence interval	
				Lower	Upper
*Model 1*					
Conventional medication cost		0.222	0.002	0.086	0.358
ALOS		0.028	< 0.001	0.013	0.043
Age		0.023	< 0.001	0.013	0.033
Sex	Female	-0.060	0.773	-0.470	0.350
Insurance type	URBMI	-0.519	0.047	-1.031	-0.008
No of hospitalizations		-0.105	< 0.001	-0.163	-0.047
Hospital type	Secondary	-0.483	0.325	-1.446	0.481
	Tertiary	-0.190	0.693	-1.135	0.755
Region	Central	-0.054	0.869	-0.704	0.595
	West	-0.253	0.292	-0.725	0.219
No of comorbidities		-0.152	0.211	-0.391	0.087
Year	2011	-0.150	0.804	-1.342	1.041
	2012	0.157	0.776	-0.928	1.242
	2013	0.121	0.821	-0.932	1.174
	2014	-0.180	0.756	-1.319	0.959
	2015	0.241	0.662	-0.846	1.328
	2016	-0.133	0.813	-1.243	0.976
Type of city	Prefecture-level city	-0.264	0.765	-2.006	1.478
	Provincial capital city	-0.365	0.680	-2.108	1.377
	Municipality	-0.020	0.984	-1.911	1.871
_Cons		3.734	< 0.001	1.683	5.784
*Model 2*					
Nonpharmacy cost		0.375	0.001	0.146	0.605
ALOS		0.031	< 0.001	0.014	0.048
Age		0.022	< 0.001	0.012	0.032
Sex	Female	-0.200	0.332	-0.607	0.206
Insurance type	URBMI	-0.586	0.024	-1.093	-0.079
No of hospitalizations		-0.100	0.001	-0.156	-0.044
Hospital type	Secondary	-0.520	0.281	-1.469	0.429
	Tertiary	-0.232	0.624	-1.164	0.700
Region	Central	0.017	0.960	-0.641	0.674
	West	-0.179	0.451	-0.648	0.289
No of comorbidities		-0.221	0.065	-0.456	0.013
Year	2011	-0.241	0.685	-1.413	0.930
	2012	0.182	0.736	-0.883	1.248
	2013	-0.026	0.962	-1.092	1.040
	2014	-0.487	0.414	-1.661	0.686
	2015	0.339	0.527	-0.717	1.395
	2016	-0.467	0.412	-1.585	0.652
Type of city	Prefecture-level city	0.170	0.844	-1.533	1.874
	Provincial Capital city	0.170	0.845	-1.540	1.879
	Municipality	0.269	0.774	-1.578	2.116
_Cons		1.985	0.125	-0.557	4.526

^a^ R-square = 0.3953 and adjusted R-square = 0.3351 / R-square = 0.4188 and adjusted R-square = 0.3604. The baseline represents the inpatient cost for a male who did not use any TCM with Urban Employee Basic Medical Insurance admitted to a primary hospital in a county-level city in the eastern region without any comorbidity. TCM: traditional Chinese medicine; UEBMI: Urban Employee Basic Medical Insurance scheme; URBMI: Urban Resident Basic Medical Insurance scheme; Municipality: municipality directly under the Central Government (i.e.Beijing, Shanghai, Tianjin, and Chongqing); ALOS: Average length of stay

## Discussion

To our knowledge, this is the first national study in China based on Medicare data to examine the economic influence of using TCM on thalassemia inpatients, and to analyze the reason of the influence. A nationally representative sample of the Chinese mainland population was used to ensure robust estimations of the cost to inpatients with thalassemia. We found that TCM users had 67.4% higher hospitalization costs than TCM nonusers, but the use of TCM was not the main reason for higher hospitalization costs.

In general, patients with minor thalassemia are in a mild condition and do not need to be hospitalized, but patients with moderate to severe thalassemia require hospitalization. After a long time of therapy, those severe thalassemia inpatients might seek alternative treatments to alleviate their pain, and TCM would be their best choice in China. Previous studies have shown that TCM is effective in relieving the symptoms of thalassemia [18–20]. We found that 37.8% of thalassemia inpatients chose to use one or more TCM approaches to treat their diseases while in hospital, which indicates that TCM has a certain degree of trust, objective demand rate, and application space, and has a good mass base in the treatment of inpatients with thalassemia in mainland China.

We found that the cost of TCM users is significantly higher than that of TCM nonusers. The TCM users have to burden 67.4% higher medical costs than TCM nonusers with other confounding factors fixed. Previous studies had shown that complementary and alternative medicine is cheaper than conventional medicines [24–26], and using TCM might alleviate the economic burdens of patients, which seems to be contrary to our results. Hence, we examined why TCM users have to burden more than TCM nonusers. From the results of the composition of medical costs in Table 4, we found that the conventional medication cost and nonpharmacy cost of TCM users accounted for the majority of the total medical cost (95.6%). The median cost of conventional medication (RMB2,259/USD340) for TCM users was significantly higher than TCM nonusers (RMB469/USD71). The median nonpharmacy cost of TCM users, such as medical treatment fees, blood transfusion fees, examination fees, and other costs (RMB5,966/USD898), were also higher than that of TCM nonusers (RMB1,304/USD196). Hence, we conclude higher conventional medication and nonpharmacy cost is the major cause of higher total inpatient costs for TCM users. The cost of TCM only accounts for a small proportion of the total medical costs and has less impact on the total medical costs. This is consistent with the results of previous studies. Huang et al. concluded that medical costs for hospitalized TCM users were 32.5% higher than for TCM nonusers, and that the cost of TCM increased significantly with increasing prescriptions of conventional medicine, which suggesting that TCM treatments were complements, rather than substitutes, to conventional treatment [22]. Nie et al. concluded that medication costs, conventional medication costs and nonpharmacy costs for TCM users were higher than for TCM nonusers, revealing that the higher medical costs for TCM users were not just to do with TCM treatments [27]. Four reasons could contribute to explaining this phenomenon.

First of all, the disease severity of TCM users might be more serious than that of TCM nonusers. Although we used the number of comorbidities as a control variable when comparing the inpatient cost of TCM users with nonusers, we also noted that the number of comorbidities was insufficient to represent the severity of the disease. In fact, the clinical outcome indicators were not included in the medical insurance database in China. When all kinds of inpatient costs for TCM users grow together, it could indicate a more serious disease situation. Patients with serious illnesses need to consume more drugs and treatment, and the serious illness will lead to a high cost of hospitalization. Second, it may also be related to people’s medical behaviors. Chinese people have a preference for the TCM culture, they believe that TCM is cheap and has few side effects [28–30]. TCM is used to choosing as an auxiliary treatment when treating diseases [31, 32]. The use of TCM is an additional treatment based on conventional treatment, so TCM cost becomes an additional financial part, and the cost of TCM users is higher than TCM nonusers. Third, after adjusting the confounding factors, we found that TCM cost was positively correlated with conventional medication and nonpharmacy cost. The increase of TCM cost is accompanied by the increase of conventional medication cost and nonpharmacy cost, which do not offset each other. This may have to do with the prescribing behavior of physicians. Doctors who are used to prescribing more western medicines also tend to prescribe more TCM, which might result in synchronous growth of both TCM costs and other costs. Last but not least, TCM plays a more complementary role but not an alternative role in the treatment of thalassemia due to the lack of combined Chinese and Western medical treatment guidelines for thalassemia diseases. In China, there are only clinical guidelines for the use of conventional treatments for thalassemia [33, 34], no guidelines for the use of TCM methods of treatment, much less for the combination of TCM and Western medicine for treatment. In clinical practice, there is no pathway for combining TCM and Western medicine, so it is not possible to achieve an alternative with better efficacy for both sides, and there is no alternative application of synergistic means, which leads to high treatment costs. Previous studies have shown that the combination of Chinese and Western medicine is clinically effective in treating diseases and can reduce the economic burden of patients [35–37], such as the treatment of acute cerebral infarction and rheumatoid arthritis. A study of hospitalization expenses for patients with acute cerebral infarction found that the cost of combined Chinese and Western medical treatment was superior to Western medical treatment, meaning that medical costs were lower for TCM users than for TCM nonusers [36]. The above may also explain the preference of some physicians to use both TCM and Western medicine. However, in the absence of a comprehensive Chinese and Western medicine treatment plan for thalassemia, doctors cannot refer to the corresponding clinical guidelines and can only prescribe based on their own experience, which is subjective and insufficient on the control over the dosage of drugs and non-drug means. Conventional methods still play a dominant role in treatment, and the addition of TCM could not balance the relationship with conventional drugs, and the arbitrary prescription of drugs might even increase the financial burdens of patients. Jianying Pan randomly divided cirrhotic ascites patients into an integrated Traditional Chinese and western medicine group and a conventional medicine group and compared the two groups and found that the cost of the integrated Chinese and Western medicine group (RMB11,570) was significantly lower than that of Western medicine group (RMB15,843.33) (p < 0.05) [38]. Gu et al. conducted a health economics evaluation of integrated traditional Chinese and Western medical for patients with Knee Osteoarthritis, Cervical Spondylosis and Lumbar Disc Herniation in a community health services center and found that integrated traditional Chinese and Western medical was more cost-effective compared to Chinese medical treatment alone [39]. Therefore, we believe that the development of integrated Chinese and Western medicine medical guidelines could have a high potential to reduce total medical costs. We urgently need a strategy for combining Chinese and Western medicine in the treatment of thalassemia to balance the use of TCM and conventional medicine, reduce patients’ expenditures, and improve treatment efficiency and clinical outcomes.

Thalassemia is often regarded as a rare disease in Chinese folk due to its limited treatment and area of occurrence. In recent years, the Chinese government has paid more and more attention to rare diseases and issued documents such as *the Catalog of the First Group of Rare Diseases* and *Guidelines for the Diagnosis and Treatment of Rare Diseases*. In 2019, the National Health Commission of the People’s Republic of China selected 324 hospitals with strong diagnosis and treatment capabilities and more cases of rare diseases to jointly establish a National Rare Disease Diagnosis and Treatment Cooperation Network to improve China’s comprehensive diagnosis and treatment capacity for rare diseases. In order to control medical costs and respond to the national policy of “attaching equal importance to both traditional Chinese medicine and Western medicine”, healthcare providers should consider the wider use of TCM, balance the relationship between TCM and Western medicine, and develop detailed guidelines for the combination of TCM and conventional medicine to treat thalassemia.

This study has several limitations. First, the study was limited by a small sample size. Rural residents, patients using only over-the-counter medications, and patients not receiving treatment from a healthcare provider were excluded. Second, due to lack of clinical indicators in the database, so the number of comorbidities was used to replace disease severity and the percentage of TCM cost to substitute the degree of TCM use. The limitation of the anonymous database prevented us from obtaining more personal information about the patients such as the patient’s income status. Third, although as many control variables as possible were included, there were significant differences in basic information between the two groups of patients, which might have influenced our findings. Fourth, we only analyzed the cost of TCM, and the nonpharmacy cost services of TCM such as acupuncture, moxibustion, massage, etc. were not included. Finally, our study only covered 2010–2016, and the post-2016 healthcare reforms may affect subsequent outcomes.

## Conclusions

Total hospitalization expenses for TCM users were higher than TCM nonusers. The proportion of conventional medication cost and nonpharmacy cost of TCM users in total inpatient medical cost were as high as 95.6%, indicating that the use of TCM is not the only reason for the high cost of TCM users. Conventional medication cost and nonpharmacy cost were positively correlated with TCM cost. We infer that TCM mainly plays a complementary role but not an alternative role in the treatment of thalassemia due to the lack of integrated Chinese and Western medical treatment guidelines. It is suggested that relevant departments should formulate diagnosis and treatment guidelines for cooperative Chinese and Western medicine treatment, and balance the use of TCM and conventional medicine, so as to reduce the economic burdens on patients.

## Electronic supplementary material

Below is the link to the electronic supplementary material.

Supplementary Material 1

## Data Availability

The data that support the findings of this study are available from China Health Insurance Research Association but restrictions apply to the availability of these data, which were used under license for the current study, and so are not publicly available. Data are however available from the author (contact Zhaoran Han) upon reasonable request and with permission of China Health Insurance Research Association.

## Abbreviations

TM: Traditional medicine
TCM: Traditional Chinese medicine
DALYs: Disability-adjusted life-years
CAM: Complementary and alternative medicine
CHIRA: China Health Insurance Research Association
UEBMI: Urban Employee Basic Medical Insurance
URBMI: Urban Resident Basic Medical Insurance
ALOS: Average length of stay
IQR: Interquartile range

## References

[1] Muncie HL, Campbell J (2009). Alpha and beta thalassemia. Am Family Phys.

[2] Alshamsi S, Hamidi S, Narci HO (2022). Healthcare resource utilization and direct costs of transfusion-dependent thalassemia patients in Dubai, United Arab Emirates: a retrospective cost-of-illness study. BMC Health Serv Res.

[3] Weatherall DJ (2018). The evolving spectrum of the epidemiology of thalassemia. Hematol Oncol Clin N Am.

[4] Taher AT, Weatherall DJ, Cappellini MD, Thalassaemia (2018). The Lancet.

[5] Weatherall DJ (2010). The inherited diseases of hemoglobin are an emerging global health burden. Blood.

[6] DALYs GBD, Collaborators H. Global, regional, and national disability-adjusted life-years (DALYs) for 359 diseases and injuries and healthy life expectancy (HALE) for 1 95 countries and territories, 1990–2017: a systematic analysis for the global burden of Disease Study 2017. Lancet.392(10159):1859–922. 10.1016/S0140-6736(18)32335-3 PMC6252083 30415748

[7] Hisam A, Khan NUS, Tariq NA, Irfan H, Arif B, Noor M (2018). Perceived stress and monetary burden among thalassemia patients and their caregivers. Pakistan J Med Sci.

[8] Zhu Y, Shen N, Wang X, Xiao J, Lu Y (2020). Alpha and beta-thalassemia mutations in Hubei area of China. BMC Med Genet.

[9] Lai K, Huang G, Su L, He Y. The prevalence of thalassemia in mainland China: evidence from epidemiological surveys. Sci Rep. 2017;7(1). 10.1038/s41598-017-00967-2 PMC5430438 28424478

[10] Lin H, Peng W, Ma Y, Miao H, Li B, Yin A (2015). Analysis of economic burden of major and intermedia thalassaemia in Guangdong province. J Med Postgraduates.

[11] Wu B, Luo R, Cao J, Chen Y, Zhang F, Qiu N (2017). The current status and strategies of medical security of patients with rare diseases in Fujian. Chin Health Econ.

[12] Mohamed SY (2017). Thalassemia major: transplantation or transfusion and chelation. Hematol Oncol Stem Cell Ther.

[13] Bordbar M, Pasalar M, Safaei S, Kamfiroozi R, Zareifar S, Zekavat O (2018). Complementary and alternative medicine use in thalassemia patients in Shiraz, southern Iran: a cross-sectional study. J Traditional Complement Med.

[14] Efe E, Isler A, Sarvan S, Baser H, Yesilipek A (2013). Complementary and alternative medicine use in children with thalassaemia. J Clin Nurs.

[15] Ismail WI, Ahmad Hassali MA, Farooqui M, Saleem F, Roslan MNF (2018). Complementary and alternative medicine (CAM) disclosure to health care providers: a qualitative insight from malaysian thalassemia patients. Complement Ther Clin Pract.

[16] Farooqui M, Hassali MA, Shatar AK, Farooqui MA, Saleem F, Haq NU (2016). Use of complementary and alternative medicines among malaysian cancer patients: a descriptive study. J Traditional Complement Med.

[17] Liao Y-H, Lin C-C, Li T-C, Lin J-G (2012). Utilization pattern of traditional chinese medicine for liver cancer patients in Taiwan. BMC Complement Altern Med.

[18] Fang S, Wu Z, Zhang X, Liu Y, Wang W, Chai L (2007). Clinical observation on YiSuiShengXueGranule on treating 156 patients with beta-thalassemia major and the molecular mechanism study. Biol Pharm Bull.

[19] Cheng YL, Zhang XH, Sun YW, Wang WJ, Fang SP, Wu ZK (2016). Clinical effect and mechanism of Yisui Shengxue granules in thalassemia patients with mild, moderate, or severe anemia. Evidence-based Complement Altern Med.

[20] Gu K, Cheng Y, Sun Y, He L, Wu Z (2021). Discussion on syndrome differentiation and treatment of thalassemia based on the theory of “kidney stores essence and produces marrow. J Tradit Chin Med.

[21] Lin SK, Lo PC, Chen WC, Lai JN (2019). Integrating traditional chinese medicine healthcare into dementia care plan by reducing the need for special nursing care and medical expenses. Med (Baltim).

[22] Huang Z, Shi X, Nicholas S, Maitland E, Yang Y, Zhao W (2021). Use of traditional chinese medicine and its impact on medical cost among urban ischemic stroke inpatients in China: a national cross-sectional study. Evidence-based Complement Altern Med.

[23] Fang H, Eggleston K, Hanson K, Wu M (2019). Enhancing financial protection under China’s social health insurance to achieve universal health coverage. BMJ.

[24] Zhai H, Chen S, Lu Y (2015). Some chinese folk prescriptions for wind-cold type common cold. J Traditional Complement Med.

[25] Timis TL, Florian IA, Mitrea DR, Orasan R. Mind-body interventions as alternative and complementary therapies for psoriasis: a systematic review of the English literature. Med (Kaunas). 2021;57(5). 10.3390/medicina57050410 PMC8146919 33922733

[26] Sarnat RL, Winterstein J (2004). Clinical and cost outcomes of an integrative medicine IPA. J Manip Physiol Ther.

[27] Nie H, Han Z, Nicholas S, Maitland E, Huang Z, Chen S et al. Costs of traditional chinese medicine treatment for inpatients with lung cancer in China: a national study. BMC Complement Med Ther. 2023;23(1). 10.1186/s12906-022-03819-3 PMC9827714 36624405

[28] Pang B, Zhao L-H, Zhou Q, Zhao T-Y, Wang H, Gu C-J (2015). Application of berberine on treating type 2 diabetes mellitus. Int J Endocrinol.

[29] Wang K, Chen Q, Shao Y, Yin S, Liu C, Liu Y (2021). Anticancer activities of TCM and their active components against tumor metastasis. Biomed Pharmacother.

[30] Guo XY, Liu J, Liu J, Li HJ, Qi Y, Qin LP (2013). Use of traditional chinese medicine in chinese patients with coronary heart disease. Biomed Environ Sci.

[31] Zhang X, Qiu H, Li C, Cai P, Qi F (2021). The positive role of traditional chinese medicine as an adjunctive the rapy for cancer. Biosci Trends.

[32] Qi F, Zhao L, Zhou A, Zhang B, Li A, Wang Z (2015). The advantages of using traditional chinese medicine as an adjunctive therapy in the whole course of cancer treatment instead of only terminal stage of cancer. Biosci Trends.

[33] Shang X, Zhang X, Yang F, Xu X (2020). Clinical practice guidelines for alpha-thalassemia. Chin J Med Genet.

[34] Shang X, Wu X, Zhang X, Feng X, Xu X (2020). Clinical practice guidelines for beta-thalassemia. Chin J Med Genet.

[35] Zhou X, Xu S, Ren Q, Chen J (2020). Quality and specific concerns of clinical guidelines for integrated chinese and western medicine: a critical appraisal. Evidence-based Complement Altern Med.

[36] Gu S, Xu X, Yue Q, Wan X (2020). Influence factors of hospitalization expenses of patients with acute cerebral infarction in different treatment routes. J Jiangxi Univ Chin Med.

[37] Xing Q, Fu L, Yu Z, Zhou X (2020). Efficacy and safety of integrated traditional chinese medicine and western medicine on the treatment of rheumatoid arthritis: a meta-analysis. Evidence-based Complement Altern Med.

[38] Pan J (2022). Analysis of the intervention effect of integrated chinese and western medical care on patients with cirrhotic ascites. Med Forum.

[39] Gu L, Jiang C, Fan C (2018). Health economics evaluation on rehabilitation mode of integrated traditional chinese and western medicine applied in community. Prev Med.

