# Supplementary material for: Differences in medical costs between TCM users and TCM nonusers in inpatients with thalassemia
Source: BMC Health Serv Res. 2023 Jun 14;23:635. doi: 10.1186/s12913-023-09651-w (PMC10268416; doi:10.1186/s12913-023-09651-w)
Supplement: Supplementary file 1 — Supplementary Material 1 [file 12913_2023_9651_MOESM1_ESM.doc]

**Additional File 1: Terminology explanation**

·Chinese herbal medicine:

Chinese herbal medicine refers to the medicinal plants produced in specific natural conditions and ecological environment, which can be used to treat and prevent diseases under the guidance of the theory of traditional Chinese medicine.

·Chinese patent medicine:

Chinese patent medicine refers to the Chinese medicine preparations which are made under the guidance of the theory of traditional Chinese medicine, via medical and pharmacological research, approved by the National Medical Products Administration, based on traditional Chinese medicine prescriptions, using herbal decoction pieces as raw materials, in accordance with the prescribed production process and quality standards.

·Chinese medicine injection:

Chinese medicine injection refers to solution, sterilized powder that can be prepared into a solution before use, or suspension, which are made by the effective substances extracted from the single side or compound of traditional Chinese medicine and natural medicine, with the use of modern pharmacy techniques and methods and under the guidance of the theory of traditional Chinese medicine, can be injected into the human body.
